# Clinical validity of clinical treatment score 5 (CTS5) for estimating risk of late recurrence in unselected, non-trial patients with early oestrogen receptor-positive breast cancer

**DOI:** 10.1007/s10549-020-06013-6

**Published:** 2020-11-21

**Authors:** Juliet Richman, Alistair Ring, Mitch Dowsett, Ivana Sestak

**Affiliations:** 1 Ralph Lauren Centre for Breast Cancer Research, Royal Marsden Hospital NHS Foundation Trust, London, UK; 2 Breast Unit, Royal Marsden Hospital NHS Foundation Trust, London, UK; 3 Centre for Cancer Prevention, Wolfson Institute of Preventive Medicine, Queen Mary University of London, London, UK

**Keywords:** Breast cancer, Extended endocrine therapy, Prediction, Late metastasis

## Abstract

**Abstract:**

**Purpose:**

Clinical Treatment Score at 5 years (CTS5) is a prognostic tool to estimate distant recurrence (DR) risk after 5 years of endocrine therapy for postmenopausal women with oestrogen receptor-positive (ER-positive) breast cancer.

**Methods:**

The validity of CTS5 was tested in a retrospective cohort of patients diagnosed with early ER-positive breast cancer. The primary endpoint was DR in years 5–10. The primary analysis cohort consisted of postmenopausal women, with premenopausal women as a secondary analysis cohort. Cox regression models were used to determine the prognostic value of CTS5 and Kaplan–Meier curves were used with associated 10-year DR risks (%).

**Results:**

2428 women were included with a median follow-up of 13.4 years. The CTS5 was significantly prognostic in both postmenopausal (*N* = 1662, HR = 2.18 95% CI (1.78–2.67)) and premenopausal women (*N* = 766, HR = 1.84 95% CI (1.32–2.56)). The 10-year DR risks were 2.9% (1.9–4.5), 7.2% (5.3–9.9), and 12.9% (10.0–16.7) for low, intermediate and high risk in postmenopausal women and 3.8% (2.2–6.7), 6.9% (4.4–10.8), and 11.1% (7.4–16.5) in premenopausal women, respectively. The number of observed DRs was significantly greater than expected in those predicted to be at high risk by CTS5 but this discordance was lost when those receiving more than 60 months of endocrine therapy were excluded.

**Conclusions:**

The CTS5 demonstrated clinical validity for predicting late DR within a large cohort of unselected postmenopausal patients but less so in premenopausal patients. Calibration of the CTS5 was good in patients who did not receive extended endocrine therapy. The CTS5 low-risk cohort has risk of DR so low as to not warrant extended endocrine therapy.

**Electronic supplementary material:**

The online version of this article (10.1007/s10549-020-06013-6) contains supplementary material, which is available to authorized users.

## Introduction

Oestrogen receptor (ER)-positive breast cancer can recur at distant sites up to at least 20 years after diagnosis [1]. Based on evidence from adjuvant studies [2–6], some women are recommended to extend endocrine therapy from 5 to 10 years instead of 5 in order to ameliorate this risk. For women who have had 5 years of an aromatase inhibitor (AI) upfront, the benefit of a further 5 years is modest [5, 7]. For this reason, identification of women who can safely avoid extended endocrine therapy is of high clinical value.

The Clinical Treatment Score at 5 Years (CTS5) is the only risk prediction model that is calibrated for postmenopausal women specifically for the risk of late distant recurrence (DR) (beyond 5 years). It is calculated from routinely collected clinicopathological variables (age, tumour size, tumour grade and lymph node burden) and was trained and validated on the datasets of postmenopausal women from the ATAC and BIG 1–98 adjuvant endocrine therapy trials [8]. These data showed the prognostic value of the CTS5 in a combined population of over 13,000 women and demonstrated its ability to assign women into specific risk categories, the lowest of which had a 3.6% risk of DR in years 5–10 and hence these women can be advised of the limited value of extended endocrine therapy.

We aimed to establish whether the prognostic value of CTS5 can be extrapolated to a ‘real-world’ cohort of women, including both post- and premenopausal women. We conducted a retrospective cohort study to test the validity of the combined ATAC/BIG 1–98 CTS5 algorithm in women treated for ER-positive early breast cancer in a single specialist centre. The result would indicate the degree of confidence with which the CTS5 may be applied in routine clinical practice and also its potential for application in premenopausal women. The CTS5 could help guide extended endocrine therapy decision making at 5 years in large numbers of women.

## Methods

### Patient cohort

**Fig. 1:**
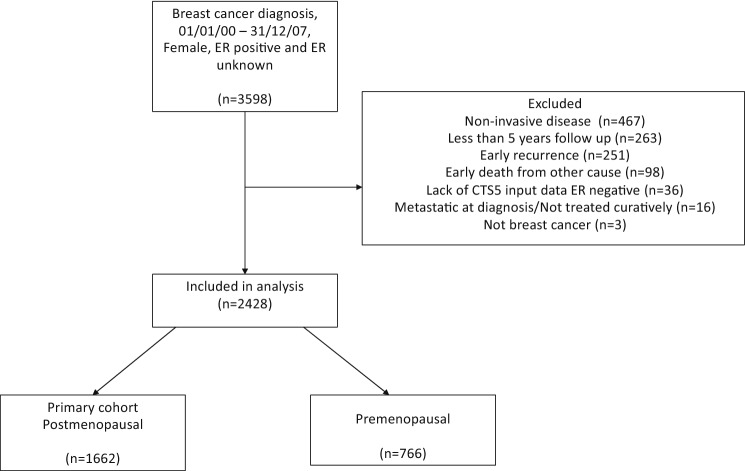
CONSORT diagram

Patients were identified through a computer-based search of hospital electronic patient records at the Royal Marsden NHS Trust. Standard demographic, histopathological, and treatment data were extracted. Inclusion criteria were: female, diagnosed with ER-positive breast cancer in years 2000–2007, and underwent surgery with curative intent. Patients were excluded if they had a non-invasive cancer pathology, less than 5 years of follow-up since diagnosis, or developed a DR within 5 years of diagnosis. Women with ER-negative disease and those with missing clinicopathological information were also excluded (Fig. 1). Women were included in this analysis regardless of HER2 status and HER2 status was recorded.

It was initially planned for endocrine therapy beyond 5 years to be an exclusion criterion. However, it was noted that by excluding these women, the cohort would become skewed in favour of those with lower stage disease. It was therefore decided that the primary analysis should include all women, irrespective of duration of endocrine therapy and a secondary analysis would exclude those receiving more than 5 years of endocrine therapy.

The CTS5 value was calculated for each patient using the published algorithm [8]. The CTS5 calculator is freely available on the internet to use (www.cts5-calculator.com). Where patients received neo-adjuvant chemotherapy or endocrine therapy, the higher of the pre-treatment and pathological tumour indices was used. For patients with less than 10 years of documented follow-up, it was assumed that they were recurrence-free.

### Statistical analysis

The primary cohort for analysis was postmenopausal women and the secondary cohort was premenopausal women. The primary endpoint was DR in years 5–10 and the secondary endpoint was DR in years 5 onwards. Local, loco-regional relapse, and contralateral new primary events in years 0–5 were not excluded. Pre-planned subgroup analyses were performed for chemotherapy-treated versus chemotherapy-untreated patients and for those who received less than or equal to 5 years of endocrine therapy.

For each endpoint, the prognostic value of the continuous CTS5 score was calculated using hazard ratios (HRs) for a unit increase in score along with 95% confidence intervals and likelihood ratios. Log-rank *P* values are presented to demonstrate degree of statistical significance. Patients were stratified into risk groups using the CTS5 cut-offs (<3.13, 3.13–3.86 and >3.86) established in the development dataset which sought to define a low, intermediate and high-risk group with 5–10-year DR risk of <5%, 5–10% and >10%, respectively. Kaplan–Meier survival method was used to obtain the survival curves for the three risk categories based on the observed distant recurrence time and events. The 5–10-year DR risks (%) were calculated with corresponding 95% confidence interval. HRs between low/intermediate and low/high-risk groups were calculated with corresponding 95% confidence intervals to demonstrate discrimination between risk groups.

Calibration of the CTS5 risk model in the postmenopausal cohort was tested by comparing observed (O) with expected (E) DR events with the dataset divided into quintiles. Expected events for each individual were derived from the baseline hazard in the original ATAC/BIG1–98 dataset. The baseline hazard was adjusted for patients with recurrences in years 5–10 and those who died of non-breast cancer causes in years 5–10. The adjustment was made on the basis of length of follow-up beyond 5 years. The overall performance of the CTS5 is shown by likelihood ratio χ^2^ test.

## Results

A total of 3598 women were identified by the electronic patient record search. After exclusions, a total of 2428 women were included in the analysis (Fig. 1), with a median follow-up of 13.4 years (IQR 11.4–15.7) from diagnosis. 1995 (82.2%) of women had HER2-negative disease, 222 (9.4%), HER2-positive disease and for 211 (8.7%) HER2 status was unknown. No post hoc analyses of the HER2-positive subgroup were therefore considered.

The population included 1662 postmenopausal women (68.5%) and 766 premenopausal women (31.5%) (Table 1). Within the postmenopausal cohort, 46% were known progesterone receptor (PR)-positive (44% were PR unknown). Median tumour size was 18 mm and 73% of cancers were node-negative. 28% of women received chemotherapy (Table 1). Within the premenopausal cohort, 54% were known PR-positive (40% PR unknown) Median tumour size was 22 mm and 60% of cancers were node-negative. 71% of women received chemotherapy. Median CTS5 score was near identical at 3.31 and 3.32 for pre- and postmenopausal women, respectively (Table 1), which translated to a similar median DR risk of 5.96% and 6.03%, respectively. Median follow-up is shown in Table 1. 337 women had a documented follow-up of less than 10 years. Throughout the follow-up period, 149 (9%) DRs were recorded in postmenopausal women, and 94 (12.3%) DRs in premenopausal women (Table 1 and Supplementary Table 1).

**Table 1:** Baseline characteristics of the pre- and postmenopausal populations

	Premenopausal (*N* = 766)	Postmenopausal (*N* = 1662)
Median age (IQR)	46 (41–49)	62.5 (57–70)
Receptor Status		
ER-Positive	766 (100%)	1662 (100%)
PR-Positive	417 (54.4%)	765 (46.0%)
PR-Negative	49 (6.4%)	173 (10.4%)
PR unknown	300 (39.2%)	724 (43.6%)
HER2-Positive	97 (12.7%)	125 (7.5%)
HER2-Negative	612 (79.9%)	1383 (83.2%)
HER2 unknown	57 (7.4%)	154 (9.3%)
Tumour size (mm), median (IQR)	22 (14–33)	18 (12–27)
Grade		
Well	105 (13.7%)	356 (21.4%)
Intermediate	405 (52.9%)	919 (55.3%)
Poor	256 (33.4%)	387 (23.3%)
Nodes		
Negative	459 (59.9%)	1211 (72.9%)
1 positive	133 (17.4%)	201 (12.1%)
2–3 positive	96 (12.5%)	134 (8.1%)
4–9 positive	64 (8.4%)	77 (4.6%)
9+ positive	14 (1.8%)	39 (2.4%)
Chemotherapy	540 (70.5%)	468 (28.2%)
Endocrine therapy		
≤ 60 months	572 (74.7%)	1405 (84.5%)
>60 months	194 (25.3%)	257 (15.5%)
CTS5, median (IQR)	3.31 (2.69–3.85)	3.32 (2.71–3.85)
Number of DR after 5 years	94 (12.3%)	149 (9.0%)
Deaths after 5 years	68 (8.9%)	388 (23.4%)
Follow-up time, median (IQR)	13.8 (11.9–16.0)	13.2 (11.2–15.6)

*IQR* interquartile range, *ER* oestrogen receptor, *PR* progesterone receptor, *HER2* human epidermal growth factor 2, *mm* millimetre, *DR* distant recurrence

### Prognostic value of the CTS5 for DR between 5 and 10 years

#### Postmenopausal cohort

**Fig. 2:**
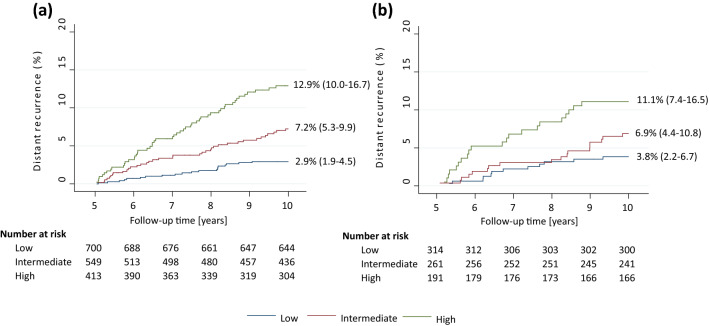
Kaplan–Meier graphs for DR according to CTS5 risk groups in (**a**) postmenopausal women and (**b**) premenopausal women

The CTS5 was significantly prognostic for late DR in years 5–10 in postmenopausal women with a HR of 2.18 ((1.78–2.67), *p* < 0.0001, LR-χ^2^ = 53.54) (Table 2). The predefined cut-off points were applied to stratify the cohort into three risk groups (Fig. 2a). Women in the intermediate or high-risk group had a significantly higher risk (HR = 2.51 ((1.46–4.33), *p* = 0.001) and 4.67 ((2.78–7.85), *p* < 0.0001), respectively) compared to those categorized into the low-risk group (Table 2). The low-risk category comprised 41% of the cohort whose risk of DR in years 5–10 was 2.9% (1.9–4.5) (Fig. 2a). The CTS5 remained highly prognostic regardless of chemotherapy treatment, with a HR of 2.08 ((2.03–3.2), *p* < 0.0001, LR-χ^2^ = 14.12) for chemotherapy-treated and HR = 2.42 (1.79–3.26), *p* < 0.0001, LR-χ^2^ = 31.06) for chemotherapy-naive patients (Table 2). We did not observe a significant interaction between CTS5 and chemotherapy use (*P* = 0.52).

**Table 2:** HRs (95% CI) for prognostic value of CTS5 according to menopausal status and follow-up period

	Years 5–10				Years 5+			
	Postmenopausal women (N = 1662)		Premenopausal women (N = 766)		Postmenopausal women (N = 1662)		Premenopausal women (N = 766)	
	HR (95% CI)	P value	HR (95% CI)	P value	HR (95% CI)	P value	HR (95% CI)	P value
CTS5 continuous	2.18 (1.78–2.67)	<0.0001	1.84 (1.32–2.56)	<0.0001	2.08 (1.74–2.47)	<0.0001	1.89 (1.48–2.41)	<0.0001
No chemotherapy	2.42 (1.79–3.26) *n* = 1194	<0.0001	1.65 (0.75–3.66) n = 226	0.21	2.27 (1.74–2.96)	<0.0001	2.42 (1.39–4.23)	0.002
Chemotherapy	2.08 (2.03–3.2) *n* = 468	<0.0001	2.21 (1.42–3.41) n = 540	<0.0001	1.82 (1.33–2.48)	<0.0001	1.85 (1.35–2.55)	<0.0001
P-interaction between CTS5 and chemotherapy		0.52		0.53		0.25		0.4
≤ 60 months ET	2.55 (2.03–3.20) *n* = 1405	<0.0001	1.83 (1.22–2.74) *n* = 572	0.003	2.29 (1.88–2.79)	<0.0001	1.81 (1.35–2.41)	<0.0001
CTS5 risk groups								
Low	Reference		Reference		Reference		Reference	
Intermediate	2.51 (1.46–4.33)	0.001	1.82 (0.88–3.78)	0.11	2.31 (1.47–3.63)	<0.0001	2.36 (1.37–4.06)	0.002
High	4.67 (2.78–7.85)	<0.0001	3.04 (1.50–6.19)	0.002	4.41 (2.89–6.74)	<0.0001	3.56 (2.06–6.13)	<0.0001

*HR* hazard ratio, *CI* confidence interval, *CTS5* clinical treatment score post 5 years, *ET* endocrine therapy

**Fig. 3:**
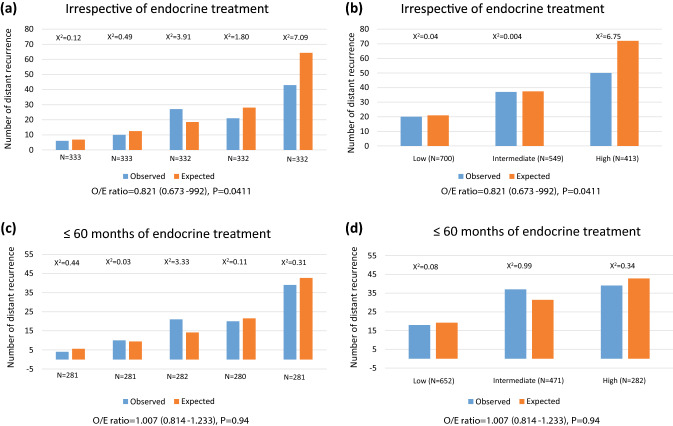
Histograms for observed versus expected events in postmenopausal women according to (**a**) quintiles and irrespective length of endocrine therapy use, (**b**) risk groups and irrespective length of endocrine therapy use, (**c**) quintiles and 60 months or less of endocrine therapy use, and (**d**) risk groups and 60 months or less of endocrine therapy use

Observed versus expected DR events showed a significant difference across quintiles with an overall χ^2^ for the difference between observed and expected of 4.2 with an O/E ratio of 0.82 (0.67–0.99) *p* = 0.041 (Fig. 3a). The significant difference was explained by discordance in the highest quintile, where significantly fewer events were observed than expected (χ^2^ = 7.09). Similarly, good observed versus expected concordance by CTS5 risk group was seen in the intermediate and low groups but lower than expected DRs were seen in the group predicted to be at high risk (Fig. 3b).

We secondarily assessed the prognostic value of the CTS5 between 5 years to end of follow-up. The continuous CTS5 had a similar HR in postmenopausal women as observed for years 5 to 10: 2.08 (1.74–2.7) versus 2.18 (Table 2). Significant risk stratification was observed between high, intermediate, and low-risk groups (high risk: HR = 4.41 (2.89–6.74), *p* < 0.0001 and intermediate risk: HR = 2.31 (1.47–3.63) *p* < 0.0001) (Table 2). Postmenopausal women in the low-risk group had a risk of DR of 6% (3.9–8.4) in years 5–19, which is approximately double compared to 5–10 years.

#### Premenopausal cohort

The CTS5 was prognostic in the premenopausal cohort in years 5–10 (HR = 1.84 (1.32–2.56), *p* < 0.0001, LR-χ^2^ = 13.25) (Table 2). In women treated with chemotherapy (*N* = 540), the CTS5 was significantly prognostic (HR = 2.21 (1.42–3.41), *p* < 0.0001, LR-χ^2^ = 12.50). For those who had not received chemotherapy (*N* = 226), the CTS5 score was not statistically significant for late DR (Table 2) but the test for interaction with chemotherapy was not statistically significant (*P* = 0.53).

**Fig. 4:**
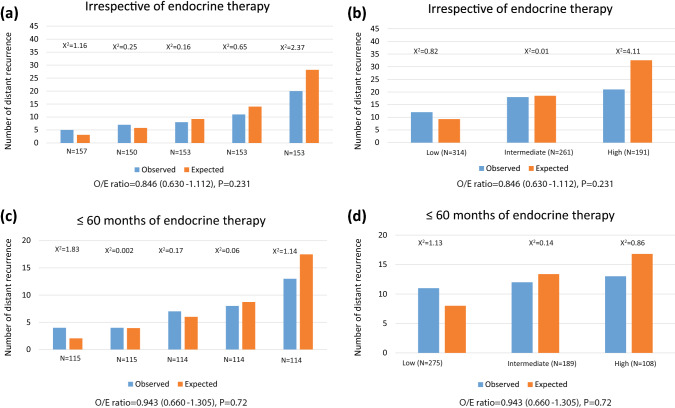
Histograms for observed versus expected events in premenopausal women according to (**a**) quintiles and irrespective length of endocrine therapy use, (**b**) risk groups and irrespective length of endocrine therapy use, (**c**) quintiles and 60 months or less of endocrine therapy use, and (**d**) risk groups and 60 months or less of endocrine therapy use

Women in the CTS5 high-risk group had a threefold increased risk of late DR compared to those in the low CTS5 risk group (HR = 3.04 (1.50–6.19), *p* = 0.002) (Table 2). The low-risk group comprised 41% of the cohort with a 5–10-year DR risk of 3.8% (2.2–6.7) (Fig. 2b). However, no significant separation between low and intermediate risk groups were observed (Table 2). Calibration of CTS5 in the premenopausal cohort showed similar numbers of observed versus expected events in all quintiles (Fig. 4a). When applying the predefined CTS5 risk groups, significantly fewer observed versus expected events were observed in the high-risk group (χ^2^ = 4.11) (Fig. 4b). Overall O/E ratio was 0.85 (0.63–1.11), *p* = 0.231.

When analysing all events from year 5 onwards in premenopausal women the continuous CTS5 had a HR for DR of 1.89 (1.48–2.41), *p* < 0.0001; LR-χ^2^ = 20.19) (Table 2). This significant effect was seen regardless of chemotherapy use. Significantly higher hazard ratios (high: HR = 3.56 (2.06–6.13), *p* < 0.0001) and intermediate: HR = 2.36 (1.37–4.06), *p* = 0.003) were observed when compared to the low-risk group (Table 2). Women who were categorized into the low-risk group had a DR risk of 9% (5.2–15.5) in years 5–19 compared with women categorized into the intermediate and high-risk groups where it was 16.5% and 25.6%, respectively.

999 women (744 postmenopausal and 255 premenopausal) had less than 10 years documented follow-up. Analysis of the whole of the pre- and postmenopausal cohorts where patients were censored at last documented follow-up showed consistent results with presumed follow-up but slightly weaker HRs owing to shorter follow-up (Supplementary Table 2).

### Prognostic value of the CTS5 for DR in those with ≤5 years of endocrine therapy

Finally, we investigated whether the length of endocrine therapy had an influence on the prediction of late DR by the CTS5. 1405 postmenopausal women and 572 premenopausal women received no more than 60 months of endocrine therapy. The prognostic performance of CTS5 was improved in postmenopausal women but was near identical to that seen in irrespective of length of endocrine therapy in premenopausal women (Table 2). The calibration in postmenopausal women was improved for the top quintile/risk group of patients such that there were no significant differences between observed and expected numbers of events (O/E ratio of 1.00 (0.81–1.23) *p* = 0.94) (Fig. 3c). Similarly, the calibration was much improved when looking at the CTS5 risk groups (Fig. 3d) with no excess predicted versus observed events in the high-risk group. In premenopausal women, the numbers of observed and expected distant recurrences were not significantly different as in the analysis irrespective of duration of endocrine therapy (Fig. 4c and d).

## Discussion

Our results support the CTS5 being highly prognostic for the prediction of late distant recurrence in postmenopausal women. This was regardless of whether women received chemotherapy or not, and similar hazard ratios were observed for events in years 5–10 and also for years 5 onwards. CTS5 stratified postmenopausal women into three distinctive risk groups and the 10-year DR risks largely mirrored those of the development set [8]. To our knowledge this is the first validation study of the combined ATAC/BIG1–98 CTS5 algorithm and it gives important, promising results. We have shown that the CTS5 extrapolates to a clinically unselected, non-trial population, is prognostic in premenopausal women and is applicable regardless of chemotherapy treatment.

Calibration of observed versus expected events was only significantly different for the top quintile of patients. Importantly, the secondary analysis, which excluded patients who were treated for longer than 60 months with endocrine therapy, led to good concordance between observed and expected events. This is almost certainly due to a reduction in risk due to extended endocrine therapy in this subgroup. Indeed, the reason for treating patients with extended endocrine treatment is to reduce their risk of relapse and the data here support that being achieved. The MA-17 study of the aromatase inhibitor letrozole showed a reduction in risk of DR of around 50% [5]. There appears to be less benefit from following 5 years of an aromatase inhibition with further aromatase inhibition but those data are less mature [9–11]. The proportions of patients receiving less than 60 months of endocrine therapy were 7%, 14% and 31% in those predicted to be at low-, intermediate- and high risk respectively, which is consistent with patients at lower risk, whose potential benefit from extended therapy is small, being denied this based on conventional risk estimates. This is also consistent with the difference in expected and observed events being most markedly changed between the primary and secondary analyses in the higher risk patients This evidence strongly supports CTS5 as an accurate prognostic tool for predicting late distant recurrence in postmenopausal women in the absence of extending endocrine therapy.

Of particular note, the CTS5 was recently reported as overestimating late distant recurrence in patients at higher risk in a combined analysis of the TEAM and IDEAL trials [12]. However, all patients in the IDEAL trial and an unreported proportion of patients in the TEAM trial received extended endocrine therapy, which would explain the apparent overestimate of risk in only the higher risk group. Clearly validity of the CTS5 for estimating risk if endocrine therapy was not extended beyond 5 years can only be fairly tested in the absence of such therapy.

Given that the CTS5 was developed in a purely postmenopausal cohort and includes an age component that reflects increased risk with age, we anticipated that its performance might be somewhat weaker in premenopausal women. The CTS5 was significantly prognostic for distant recurrence in years 5–10 and years 5 onwards in the premenopausal population. The CTS5 identified a low-risk group whose DR risk in years 5–10 was 4.9%. However, this was greater than that seen in postmenopausal women and risk group stratification was not distinctive compared to the postmenopausal cohort. Interestingly however, there was an almost doubling of DR events among premenopausal women beyond 10 years and this resulted in a more distinct separation of risk groups. Overall, the CTS5 demonstrated significant prognostic performance in premenopausal women but did not show strong clinical validity with regards risk group stratification as seen with postmenopausal women. The CTS5 might benefit from recalibration for use in premenopausal women. Of note, CTS5 was also applied to the TAILORx dataset and was found to have good prognostic value for women over 50 but, as here, less so for women under 50 in an overall low stage population [13].

Strengths of this study are that the analysis is comprised of a large number of clinically unselected women and therefore represents a ‘real-life’ cohort. Data were carefully collected by manual assessment of individual patient records after an initial computer search. Findings from the postmenopausal cohort generally mirror that of the development dataset showing consistency between populations. Limitations of the study are its retrospective nature and therefore relies on adequate documentation, which can have an impact on record-keeping. Within this study there was a small proportion of patients with less than 10 years of follow-up, such that some events may have been missed in patients who were lost to follow-up. We estimated that this number is very low given that most women who experienced a recurrence return to their treating hospital for management. As in the original development of CTS5, the current study does not address the predictive value of the CTS5 for extended endocrine therapy. Applying the score to a cohort that has been randomized to continue endocrine therapy to 10 years versus stopping at five should therefore be conducted to generate evidence for the clinical utility of CTS5. Owing to the historic nature of this cohort, we were unable to perform subgroup analyses based on HER2 subtype. However, evaluation of the CTS5 in an ER-positive/HER2-positive population that has received HER2-targeted therapy is an important research question to address given the varied data that exists on HER2 status in the context of ER-positive breast cancer [14–16].

Several commercially available genomic assays have shown prognostic value for late distant recurrence [17–20] and have the benefit of incorporating clinical parameters with molecular predictors. Molecular assays, while adding more precision to risk estimation in years 0–5, carry a high cost and burden of resources, which will preclude their use in many centres both UK and worldwide. CTS5 on the other hand represents a cost-free, accessible tool that could be implemented immediately.

We recommend the use of the CTS5 for postmenopausal women treated for ER-positive, HER2-negative breast cancer who are distant recurrence-free at 5 years. Our data supports recommendations that women who are ‘CTS5 low’ can safely stop endocrine therapy at 5 years owing to the low potential added benefit from continuing. Women who are ‘CTS5 high’ are at sufficient risk to consider that continuing endocrine therapy would likely be worthwhile. For those that fall into the intermediate category the management pathway is less clear and it may be that genomic models could further risk stratify these women.

## Electronic supplementary material

Below is the link to the electronic supplementary material.

Electronic supplementary material 1 (DOCX 28 kb)

## Data Availability

The datasets generated and/or analysed during the current study are available from the corresponding author on reasonable request.
