# Supplementary material for: Clinical validity of clinical treatment score 5 (CTS5) for estimating risk of late recurrence in unselected, non-trial patients with early oestrogen receptor-positive breast cancer
Source: Breast Cancer Res Treat. 2020 Nov 21;186(1):115–23. doi: 10.1007/s10549-020-06013-6 (PMC7940308; doi:10.1007/s10549-020-06013-6)
Supplement: Supplementary file 1 — Electronic supplementary material 1 (DOCX 28 kb) [file 10549_2020_6013_MOESM1_ESM.docx]

**Supplementary Table 1** – Numbers of patients and event numbers for total cohorts and subgroups.

|  | *Postmenopausal* | | *Premenopausal* | |
| --- | --- | --- | --- | --- |
|  | **Number** | **DR events** | **Number** | **DR events** |
| **Total** | 1662 | 149 (9.0%) | 766 | 94 (12.3%) |
| **No chemotherapy** | 1194 | 82 (6.9%) | 226 | 20 (8.9%) |
| **Chemotherapy** | 468 | 67 (14.3%) | 540 | 74 (13.7%) |
| **CTS5 Low** | 700 | 31 (4.4%) | 341 | 20 (6.4%) |
| **CTS5 Intermediate** | 549 | 49 (8.9%) | 261 | 37 (14.2%) |
| **CTS5 High** | 413 | 69 (16.7%) | 191 | 37 (19.4%) |
| **<60 months endocrine therapy** | 1405 | 128 (9.1%) | 572 | 70 (12.2%) |

**Supplementary Table 2** - Comparison of documented and assumed follow up of all a. post-menopausal and b. pre-menopausal women.

|  | **Documented follow-up** | | | **Assumed follow-up** | | | | |
| --- | --- | --- | --- | --- | --- | --- | --- | --- |
| *Post-menopausal*  *N=1662, DR=107* | | | | | | | | |
|  | **HR (95% CI)** | **P-value** | **LR-χ^2^** | **HR (95% CI)** | | **P-value** | | **LR-χ^2^** |
| **Continuous CTS5** | 1.95 (1.59-2.39) | <0.0001 | 40.10 | 2.18 (1.78-2.67) | | <0.0001 | | 53.54 |
| **Low risk (N=700 (42.1%))** | Reference |  | - | Reference | | - | | - |
| **Intermediate risk (N=549 (33.0%))** | 2.28 (1.32-3.93) | 0.003 | - | 2.51 (1.46-4.33) | | 0.001 | | - |
| **High (N=413 (24.9%))** | 3.81 (2.27-6.41) | <0.0001 | - | 4.67 (2.78-7.85) | | <0.0001 | | - |
| *Pre-menopausal*  *N=776, DR=107* | | | | | | | | |
| **Continuous CTS5** | 1.72 (1.23-2.40) | 0.001 | 10.39 | 1.84 (1.32-2.56) | <0.0001 | | 13.25 | |
| **Low risk (N=314 (41.0%))** | Reference |  | - | Reference | - | | - | |
| **Intermediate risk (N=261 (34.1%))** | 1.69 (0.84-3.51) | 0.16 | - | 1.82 (0.88-3.78) | 0.11 | | - | |
| **High (N=191 (24.9%))** | 2.63 (1.29-5.34) | 0.008 | - | 3.04 (1.50-6.19) | 0.002 | | - | |
